# Optimized induction of mitochondrial apoptosis for chemotherapy-free treatment of BCR-ABL+acute lymphoblastic leukemia

**DOI:** 10.1038/s41375-018-0315-6

**Published:** 2018-12-13

**Authors:** Michaela Scherr, Hanna Kirchhoff, Karin Battmer, Katharina Wohlan, Chun-Wei Lee, Melanie Ricke-Hoch, Sergej Erschow, Edward Law, Arnold Kloos, Michael Heuser, Arnold Ganser, Denise Hilfiker-Kleiner, Olaf Heidenreich, Matthias Eder

**Affiliations:** 1 Department of Hematology, Hemostasis, Oncology and Stem Cell Transplantation, Hannover Medical School, Hannover, Germany; 2 Department of Cardiology and Angiology, Hannover Medical School, Hannover, Germany; 3 Newcastle Cancer Centre at the Northern Institute for Cancer Research, Newcastle University, Newcastle upon Tyne, UK

**Keywords:** Acute lymphocytic leukaemia, Targeted therapies

## Abstract

BCR-ABL+acute lymphoblastic leukemia (ALL) in adults has a poor prognosis with allogeneic stem cell transplantation (SCT) considered the best curative option for suitable patients. We here characterize the curative potential of BH3-mimetics differentially targeting mitochondrial BCL2-family members using a combination therapy approach with dexamethasone and tyrosine kinase inhibitors targeting BCR-ABL. In BCR-ABL + ALL BH3-mimetics act by redistribution of mitochondrial activator BIM, which is strongly required for cytotoxicity of the BCL2-specific BH3-mimetic ABT-199, tyrosine kinase inhibitors (TKIs) and dexamethasone. BIM expression is enhanced by dexamethasone and TKIs and both synergize with ABT-199 in BCR-ABL + ALL. Triple combinations with ABT-199, dexamethasone and TKIs efficiently attenuate leukemia progression both in tissue culture and in primary cell xenotransplantation models. Notably, the dasatinib-containing combination led to treatment- and leukemia-free long-term survival in a BCR-ABL + mouse model. Finally, response to BH3-mimetics can be predicted for individual patients in a clinically relevant setting. These data demonstrate curative targeted and chemotherapy-free pharmacotherapy for BCR-ABL + ALL in a preclinical model. Clinical evaluation, in particular for patients not suitable for allogeneic SCT, is warranted.

## Introduction

Acute lymphoblastic leukemia (ALL) is a genetically heterogeneous disease with a 5-year survival of about 40% for adults. Precursor B-ALL with the Philadelphia-translocation t(9;22)(q34;q11) defines a very high-risk group [1–3] and encodes the BCR-ABL oncoprotein with constitutive tyrosine kinase activity (BCR-ABL + ALL). Targeted therapy of BCR-ABL + ALL with ABL-specific tyrosine kinase inhibitors (TKI) such as imatinib and dasatinib combined with induction and post-remission chemotherapy prolongs remission but relapse rates remain high [4]. Therefore, allogeneic hematopoietic stem cell transplantation (SCT) remains the therapy with the highest curative potential. However, SCT has high transplant-associated morbidity and mortality and is limited to younger and suitable patients [4–7]. Therefore, improvement of pharmacotherapy remains of crucial importance to improve survival of BCR-ABL + ALL patients.

Cellular apoptosis can be induced either by mitochondrial or death receptor signaling. Mitochondrial outer membrane permeabilization (MOMP) is considered the point of no return of mitochondrial apoptosis [8, 9]. MOMP releases cytochrome C into the cytosol thereby activating the apoptosome and downstream effector caspases. MOMP is controlled in part by protein-protein interactions between pro- and anti-apoptotic BCL2-family members interacting mostly via α-helical BH3 domains and BH3 domain binding grooves. MOMP is induced by transfer of BH3-only proteins BIM or BID from the anti-apoptotic factors such as BCL2, BCLXL or MCL1 to apoptotic proteins BAX and BAK. This shift is facilitated by sensitizers such as BAD, BIK, NOXA or PUMA [8, 9].

BH3 mimetics that directly target the BH3 domain binding grooves of anti-apoptotic BCL2 proteins have been developed based on affinity screens and structure-based modeling. For example, ABT-737 binds to BCL2, BCLXL and BCLW whereas ABT-199 and WEHI-539 specifically bind to BCL2 and BCLXL, respectively [10, 11]. Binding of BH3-mimetics is thought to either release BH3-only proteins and/or to prevent their binding to anti-apoptotic BCL2 proteins leading to BAK/BAX oligomerization. Recently, ABT-199 has been clinically approved as the first BH3-mimetic for the treatment of chronic lymphocytic leukemia [12, 13]. We already demonstrated efficacy of ABT-737 in a xenograft BCR-ABL + ALL model [14]. Furthermore, MCL1 inhibitors have been developed [15] and recently entered clinical trials.

We hypothesized that BCR-ABL + ALL may be amenable to targeted therapy with BH3-mimetic-containing drug combinations. We therefore biochemically examined induction of MOMP by BH3 mimetics in BCR-ABL + ALL and further explored a targeted and potentially curative chemotherapy-free combination therapy consisting of ABT-199, dexamethasone, and dasatinib in vitro and in vivo.

## Methods

### Patient material

BM and PB samples were collected from newly diagnosed BCR-ABL-positive and negative B-lineage ALL, and CML respectively. For further information see Supplementary Material.

### Animal experiments

Leukemic BV173 cells (BV173-SLIEW) (1 × 10^6^) or patient derived xenograft (PDX) cells were adoptively transplanted into recipient NSG (NOD.Cg-Prkdcscid Il2rgtm1WjI/SzJ) mice intravenously. For in vivo treatment and leukemic monitoring see Supplementary Materials.

### Short-term BH3-mimetics response assay

Ficoll gradient-purified mononuclear cells from PB from newly diagnosed B-lineage ALL, from healthy volunteers, CML CD34^+^ cells, or BV173 or SUPB15 cells were cultured at 1 × 10^6^ cells/ml in the presence of increasing concentrations of ABT-199 (0, 0.001, 0.01, 0.1, 1 µM) for 3 h followed by staining with 50 nM TMRE (tetramethylrhodamine ethyl ester) for 20 min at 37 °C. Separately, cells were treated with the ionophore FCCP (5 µM) (Carbonyl cyanide-4-(trifluoromethoxy)phenylhydrazone) as a positive control. MOMP of viable cells was determined by flow cytometry.

Pre-treatment of PDX cells co-cultured on bone marrow derived mesenchymal stem cells (MSCs) or of BV173 cells with dexamethasone (100 or 50 nM) and dasatinib (50 or 0.5 nM) was performed for 48 h prior to TMRE assay.

Cell culture, cloning of lentiviral constructs, apoptosis analysis, proliferation assays, immunoprecipitation, immunoblotting and statistical methods are described in detail in Supplementary Materials.

## Results

### Induction of mitochondrial apoptosis in BCR-ABL+ALL by BH3-mimetics

Since the specific BCL2 inhibitor ABT-199 is now approved for CLL therapy [16], we first analyzed the ABT-199 half-maximal inhibitory concentrations (IC50) in several BCR-ABL-positive and -negative ALL cell lines (Fig. S1A). The IC50s ranged from 0.002 to 2 µM demonstrating ABT-199 cytotoxicity in most ALL cell lines.

**Fig. 1:**
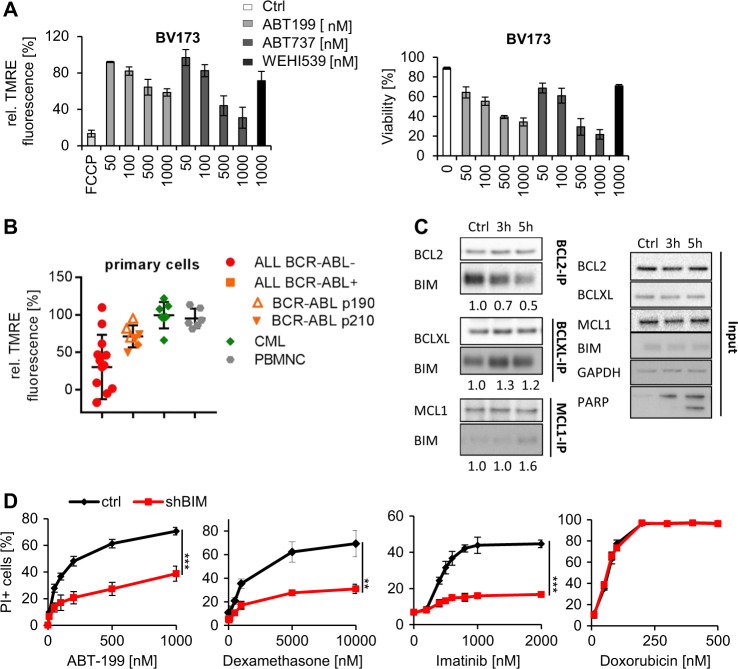
Induction of mitochondrial apoptosis in BCR-ABL + ALL by BH3-mimetics. **a** (Left) BV173 were treated with various concentrations of ABT-199, ABT-737, and WEHI-539 for 3 h followed by staining with TMRE to determine MOMP of viable cells by flow cytometry. Treatment with the ionophore FCCP served as positive control, and fluorescence of untreated cells was set as 100%. (Right) PI staining to monitor cell viability was performed by flow cytometry after additional 21 h. Values are expressed as means ± SD (*n* = 3). **b** PB mononuclear cells from newly diagnosed BCR-ABL-negative ALL (*n* = 13), BCR-ABL-positive ALL (*n* = 7), CML CD34^+^ cells (*n* = 7) and PB mononuclear cells from healthy volunteers (*n* = 6) were incubated with 1 µM ABT-199 for 3 h for MOMP induction followed by TMRE staining and FACS analysis of viable cells. *p*-value was calculated using Student’s *t*-test (*p < 0.05). **c** BV173 cells were treated with 100 nM ABT-199 for the indicated periods of time. Cellular lysates were immunoprecipitated with anti-BCL2, anti-BCLXL, and anti-MCL1 antibodies, respectively. Total cellular lysates and immunoprecipitates were subjected to western blot analysis using the indicated antibodies. Quantification of band intensities was normalized to the amount of precipitated target protein. **d** BV173 cells were stably transduced with lentiviral constructs encoding specific shRNA targeting *BIM* or a control sequence and treated with various concentrations of ABT-199, dexamethasone, imatinib or doxorubicin for 48 h. PI staining was performed to monitor cell viability. Values are expressed as means ± SD (*n* = 3). *p*-values were calculated using Student’s *t*-test (**p < 0.01, ***p < 0.001)

We next quantified MOMP induction by BH3-mimetics in viable cells using a short-term flow cytometry-based assay. Mitochondria were stained with TMRE using mitochondrial bleaching by the uncoupling agent FCCP as a positive control. ABT-199 and ABT-737 rapidly induced a decrease in TMRE fluorescence preceding apoptotic death in BCR-ABL+ cells in a dose-dependent manner (Fig. 1a, S1B). In contrast to ABT-199 and ABT-737, WEHI-539 induced less (BV173) or no (SUPB15) MOMP and apoptosis in these cells. MOMP induction highly correlated with cell viability with *r* > 0.905 and 0.997 in BV173 and SUPB15 cells, respectively (Figs. S1C, S1D).

We then used this short-term assay to analyze ABT-199 cytotoxicity in normal peripheral blood mononuclear cells (PBMNCs), mononuclear cells from newly diagnosed BCR-ABL-positive and BCR-ABL-negative B-lineage ALL patients as well as chronic phase CML CD34+ cells. Cells were incubated with 1 µM ABT-199 for 3 h followed by TMRE staining and flow-cytometric analysis of living cells. Normal PBMNCs and CML CD34+ cells show almost no MOMP induction (reduction of MFI to 95.4% and 99.6% only, respectively), whereas ALL blasts exhibited a marked reduction of TMRE fluorescence (Fig. 1b). Interestingly, BCR-ABL + primary ALL cells were more resistant against ABT-199 than BCR-ABL-negative leukemic blasts with MFI-reduction to 71.3% and 39.0%, respectively (*p* = 0.04). We did not observe a significant difference in ABT-199-mediated MOMP induction between samples with the e1-a2 (p190 BCR-ABL, *n* = 3) and the b2-a2/b3-a2 (p210 BCR-ABL, *n* = 3) isoforms. These data indicate that BCR-ABL may interfere with ABT-199 cytotoxicity in primary ALL cells.

Since BH3-mimetics act by release of MOMP activators [8, 9], we analyzed the protein expression of mitochondrial BCL2-family members in ALL cells. Expression of both anti- and pro-apoptotic BCL2 members was heterogeneous with low BIM (BCL2L11) expression in all BCR-ABL + ALL lines and patient material (Figs. S1E, S1F and S1G).

Next, we examined the binding and the redistribution of MOMP activators induced by different BH3-mimetics. In the absence of BH3-mimetics, BIM but not BID bound to BCL2 and BCLXL in BV173 and SUPB15 cells (Fig. 1c, S1H). In contrast, BIM was hardly detectable in MCL1 immunoprecipitates. ABT-199 displaced BIM from BCL2 in a time- and dose-dependent manner leading to induction of apoptosis as shown by PARP cleavage (Fig. S1I). In parallel, BIM/BCLXL and BIM/MCL1 complexes were formed (Fig. 1c, S1J). Finally, the binding profile of BH3-mimetics determined BIM redistribution to anti-apoptotic BCL2 proteins: ABT-199, WEHI-539, and ABT-737 released BIM from BCL2-only, BCLXL-only, and BCL2 and BCLXL, respectively, leading to increased BIM binding to non-targeted BCL2-family members (Figs S1J, S1K).

Dexamethasone (DEX) and BCR-ABL-specific tyrosine kinase inhibitors (TKIs) are important and crucial components of current pharmacotherapy of BCR-ABL + ALL. Since DEX and TKIs are known to enhance BIM expression in ALL cells, we proposed that these compounds might be especially useful in combination with ABT-199 in BCR-ABL + ALL. Accordingly, DEX induced BIM expression in BV173 and SUPB15 cells (Fig. S1L), and TKIs reduced BIM phosphorylation in addition to a slightly enhanced BIM expression (Fig. S1M). Furthermore, expression of BCLXL and MCL1 [17] were reduced in the presence of TKIs in BV173 and SUPB15 cells (Fig. S1M) in line with earlier reports.

To validate the functional role of BIM for cytotoxicity of BH3-mimetics, DEX, and imatinib (IM), we transduced BV173 cells with shBIM with a transduction rate of more than 90% yielding a more than threefold reductions of BIM expression (data not shown). BIM knockdown diminished cytotoxicity of ABT-199, dexamethasone and imatinib indicating that all three compounds heavily depended on BIM expression (Fig. 1d). In contrast, doxorubicin acted BIM-independently (Fig. 1d). Furthermore, anti-*BID* shRNAs had no effect on the cytotoxicity of either drug (data not shown). Thus, the cytotoxicity of ABT-199, DEX, and imatinib depends on BIM expression, suggesting that these drugs act within signaling pathways which converge on induction of mitochondrial apoptosis.

### Cooperation of ABT-199 with DEX and imatinib

**Fig. 2:**
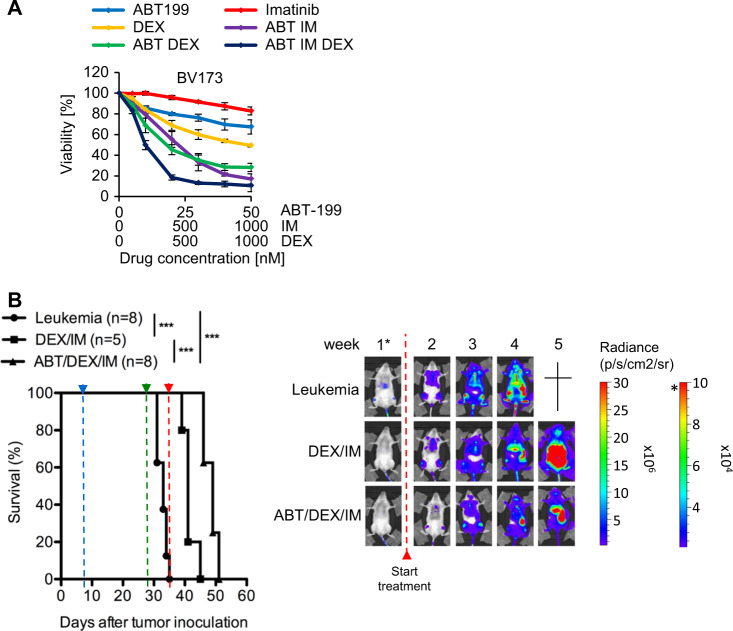
Cooperation of ABT-199 with dexamethasone and imatinib. **a** BV173 cells were treated with ABT-199, IM, DEX alone or in combination at the indicated concentrations at fixed ratios for 48 h followed by PI staining. Values are expressed as means ± SD (*n* = 3). Combination indices were calculated using the Chou Talalay method. CI values < 1 were considered as synergistic drug interaction. CI (ABT/DEX) = 0.46, CI (ABT/IM) = 0.21, CI (ABT/DEX/IM) = 0.15, **b** NSG recipients received 1 × 10^6^ BV173 cells intravenously. Tumor proliferation was monitored by using in vivo bioluminescent IVIS assay. Treatment started one week after tumor inoculation (blue line). Kaplan–Meier survival curves (left) and representative IVIS images (right) of recipients treated with DEX (1 mg/kg) and IM (20 mg/kg) or in combination with ABT (20 mg/kg) by oral gavaging 5 days per week. Begin of therapy is indicated by the blue arrowhead. Increased doses of ABT were given at week 4 (50 mg/kg) (green arrowhead) and week 5 (75 mg/kg) (red arrowhead) post tumor inoculation, respectively. Log-rank test was used for statistical survival analyses (***p < 0.001)

Since these data provide a rationale to combine DEX and imatinib with ABT-199 in BCR-ABL + ALL, we examined a potentially synergistic action of ABT-199, dexamethasone, and imatinib by dose-effect combination index (CI) analysis. In BV173 cells, ABT-199, dexamethasone, and imatinib exhibited dose-dependent cytotoxicity, and both compounds synergized with ABT-199 with CI values of 0.5 and 0.19 (values < 1 considered synergistic), respectively, whereas the triple-agent therapy synergized with CI = 0.15 (Fig. 2a). In addition, while both imatinib and DEX showed a late onset of cytotoxicity achieving its maximum after 96 h or later, the triple combination showed high efficacy already after 48 h. These data demonstrate not only synergy but also an additional kinetic benefit of the triple combination over dexamethasone or imatinib. In contrast to BV173 cells, SUPB15 cells are resistant to TKIs [18]. In agreement with this, SUPB15 cells were killed by ABT-199 and DEX but not imatinib when applied as single agents (Fig. S2). However, imatinib strongly synergized with ABT-199 and ABT-199/DEX in these cells with CIs of 0.40 and 0.03, respectively (Fig. S2).

Given the robust in vitro synergism of ABT-199, DEX, and TKIs, we analyzed drug combinations in vivo using a murine NSG xenotransplantation model with luciferase-transduced BV173 cells to allow monitoring of leukemic growth by bioluminescent imaging [19]. BV173 cells rapidly induced aggressive acute leukemia with a maximal survival of about 35 days (Fig. 2b). Treatment was initiated one week after transplantation and confirmed engraftment (detection of about 15% GFP+ cells in spleens of transplanted mice (*n* = 3 mice, range 14–17%). Mice were treated orally with vehicle alone (leukemia control), or with a combination of dexamethasone and imatinib as double-agent and triple-agent (ABT-199/DEX/IM) therapy. The ABT-199/DEX/IM combination was superior to DEX/IM double-agent therapy both in terms of tumor growth and survival (median survival 48.5 and 42 days, respectively, *p* < 0.001). However, none of the mice obtained a complete remission as detected by bioluminescence, and all mice died during therapy due to leukemic progression indicating the limitation of this combination therapy.

### Curative pharmacotherapy of BCR-ABL+ALL with ABT-199, DEX, and DAS

**Fig. 3:**
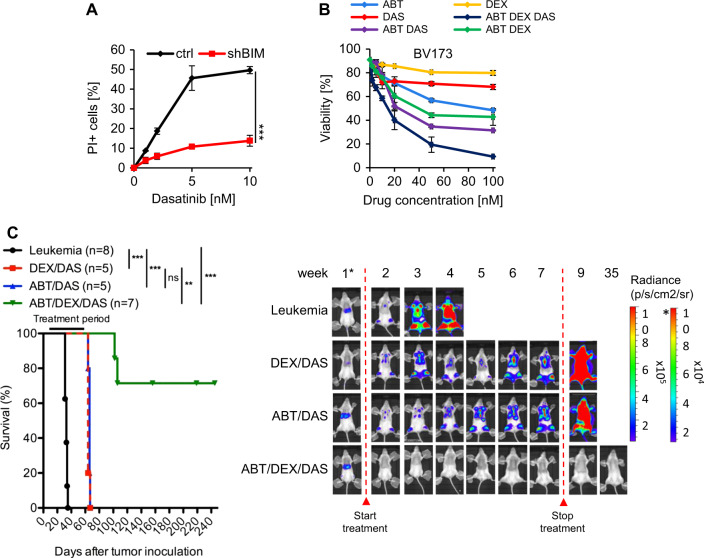
Curative pharmacotherapy of BCR-ABL + ALL with ABT-199, dexamethasone and dasatinib. **a** BV173 cells were stably transduced with lentiviral constructs encoding specific shRNA targeting *BIM* or a control sequence and treated with various concentrations of dasatinib for 48 h. PI staining was performed to monitor induction of apoptosis. Values are expressed as means ± SD (*n* = 3). *p*-values were calculated by Student’s *t*-test (***p < 0.001). **b** BV173 cells were treated with ABT-199, DAS, DEX alone or in combination at fixed ratios for 24 h followed by PI staining. Values are expressed as means ± SD (*n* = 3). Combination indices were calculated using the Chou Talalay method. CI (ABT/DEX) = 0.88, CI (ABT/DAS) = 0.62, CI (ABT/DEX/DAS) = 0.15. **c** NSG recipients received 1 × 10^6^ BV173 cells intravenously. Tumor proliferation was monitored by using in vivo bioluminescent IVIS assay. Treatment started 1 week after tumor inoculation. Kaplan–Meier survival curves (left) and representative IVIS results for week 1–35 (right) of recipients treated with DEX (1 mg/kg) and DAS (10 mg/kg) or in combination with ABT (constant dose of 20 mg/kg) by oral gavaging 5 days per week are shown. Treatment was stopped after week 7. Data are summarized from three independent experiments. Log-rank test was used for statistical survival analyses (**p ≤ 0.01, ***p ≤ 0.001)

Dasatinib is a dual ABL/SRC and second generation ABL inhibitor [20] which may be more efficient than imatinib when combined with ABT-199. Similar to imatinib, dasatinib enhanced expression of BIM and reduced expression of BCLXL and MCL1 (Fig. S3A). Furthermore, dasatinib-associated cytotoxicity heavily depended on BIM (Fig. 3a) suggesting that inhibition of SRC kinases by dasatinib may also converge on the induction of mitochondrial apoptosis. DAS acted synergistically with ABT-199 (CI = 0.62) and DEX plus ABT-199 (CI = 0.15) (Fig. 3b). Moreover and similar to the imatinib-containing triple combination, DAS/DEX/ABT showed maximal efficacy already after 24 h and, thus, substantially earlier than dasatinib and dexamethasone as single agents.

As DAS showed strong in vitro response with ABT-199 and DEX, we next sought to determine the efficacy of triple-agent therapy with DAS in vivo. ABT-199/DEX/DAS was much more efficient than DAS/DEX or ABT-199/DAS leading to a more rapid and long-lasting tumor reduction to even undetectable levels (Fig. 3c). Treatment was discontinued after 6 weeks, and five out of seven ABT-199/DEX/DAS-treated mice remained leukemia-free for the whole observation period of more than 35 weeks. In contrast, all DAS/DEX- and DAS/ABT-199-treated mice rapidly died within two weeks after the end of treatment due to progression of leukemia.

These results show superiority of DAS as compared to IM and imply that additional DAS targets beside BCR-ABL may also enhance synergy with ABT-199 and DEX.

### Modification of combination therapy by pulsatile application of ABT-199

**Fig. 4:**
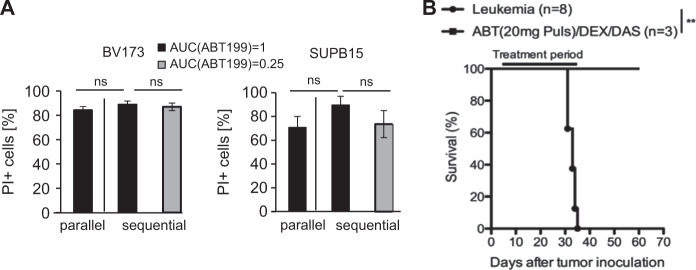
Modification of combination therapy by pulsatile application of ABT-199. **a** BV173 (left) and SUPB15 (right) cells were treated for a total of 96 h with DAS (50 and 10 nM, respectively) and DEX (50 and 10 nM, respectively). ABT-199 (50 nM in BV173 and 10 nM in SUPB15, respectively) was added either in parallel (ABT-199 AUC = 1) or as sequential treatment for the last 24 h in either equal concentration (equivalent to ABT-199 AUC = 0.25) or 4-fold higher concentration (equivalent to ABT-199 AUC = 1). Values are expressed as means ± SD (*n* = 3). *p*-values were calculated by Student’s *t*-test. **b** NSG recipients received 1 × 10^6^ BV173 cells intravenously. Treatment started 1 week after tumor inoculation. Kaplan–Meier survival curves of recipients treated with DEX (1 mg/kg) and DAS (10 mg/kg) by oral gavaging 5 days per week and pulsatile therapy with ABT-199 (constant dose of 20 mg/kg) on the 4th day (96 h) of the weekly treatment cycle are shown. Treatment was stopped after week 6. Log-rank test was used for statistical survival analyses (**p ≤ 0.01)

ABT-199 quickly (within hours) interrupted the BCL2/BIM-binding in a time- and dose-dependent manner (Fig. 1c and S1I). In contrast, the cytotoxicity of DEX and DAS required several days to enhance BIM expression (Figs. S3 and S1L). Accordingly, the kinetics of drug-induced cytotoxicity differed between ABT-199 on one side and DEX and DAS on the other (Fig. S4A). Interestingly, the triple-agent therapy combined the rapid onset of cytotoxic effects (ABT-199) with increasing efficacy over-time (DEX, DAS). We therefore hypothesized that initial BIM-preloading of BCL2 before administration of ABT-199 may provide a strategy to optimize combination therapies. In experiments with either continuous or pulsatile delayed addition of ABT-199 to DEX and DAS at doses with either equal or quarter “area under the curve” (AUC) amounts of ABT-199, the pulsatile therapy was equally effective at reduced AUC in BV173 and SUPB15 cells (Fig. 4a). In vivo, the combination therapy with continuous application of DEX/DAS and pulsatile therapy with ABT-199 on only 1 of 5 days (Fig. 4b) (ABT-199 AUC = 0.2 as compared to continuous ABT-199 application as shown in Fig. 3c) induced leukemia control even after discontinuation of therapy. However, all mice relapsed within 4 weeks after end of treatment as demonstrated by in vivo bioluminescence (Fig. S4B). These findings suggest that pulsatile ABT-199 therapy might be inferior in achieving long-term remission.

### Effects of ABT-199, DEX, and DAS on BCR-ABL+ALL primografts

**Fig. 5:**
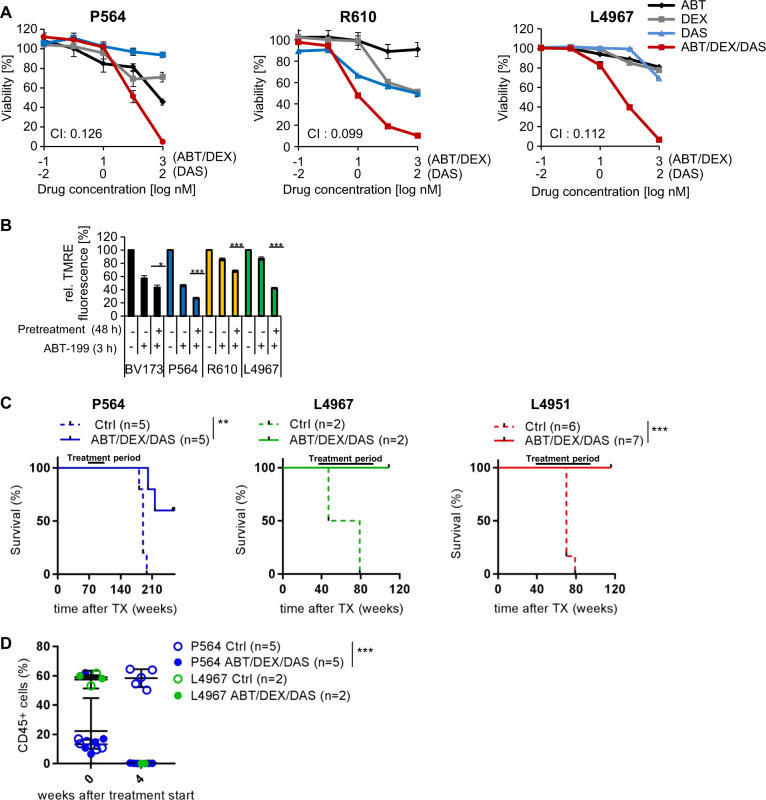
Effects of ABT-199, DEX, and DAS on BCR-ABL + ALL primografts. **a** Patient-derived P564 (left), R610 (middle), and L4967 (right) cells were plated 24 h prior to drug treatment onto primary MSCs. Co-cultures were treated with increasing concentrations of ABT-199, DEX, and DAS alone or in combination with fixed ratios. Fluorescence microscopic analysis of Calcein AM/PI/DAPI staining was performed to determine apoptotic cell death. Quantification of viable cells by counting viable and apoptotic cells in three representative microscopic pictures per well in triplicates. Values are expressed as means ± SD (*n* = 3). CI values were calculated using the Chou Talalay method. P564 CI(ABT/DEX/DAS) = 0.126; R610 CI(ABT/DEX/DAS) = 0.099. L4967 (CI/ABT/DEX/DAS) = 0.112. **b** BV173 and patient-derived P564, R610 and L4967 cells (primografts on MSCs) were pretreated with DEX and DAS for 48 h before ABT-199 application for 3 h and mitochondrial staining with TMRE. Decrease in TMRE MFI is expressed as means ± SD (*n* = 3) as compared to untreated controls set 100%. *p*-values were calculated by Student’s *t*-test (*p *<* 0.05; ***p < 0.001). **c** NSG recipients received 1 × 10^6^ P564, L4967, and L4951 PDX cells intravenously and survival of control and triple combination treated mice (ABT-199 (20 mg/kg), DEX (1 mg/kg) and DAS (10 mg/kg) by oral gavaging 5 days per week) is shown. **d** Tumor cell kinetics was monitored by human CD45 expression in peripheral blood cells (P564 and L4967). Note that all control mice engrafted with L4967 cells died before the time of analysis at 4 weeks

Having demonstrated the superiority of the ABT-199/DEX/DAS triple combination therapy to dual combinations in BV173 xenograft models we wanted to test the efficacy of this therapy more generally in patient-derived xenografts. We therefore established four primograft models from BCR-ABL + ALL patients with one of them (L4951) transduced with luciferase to allow in vivo imaging of leukemic growth. All but L4951 primograft cells could be cultured ex vivo on human bone marrow-derived MSCs [20] (Fig. S5A). In cultures of P564, R610 and L4967 cells on MSCs with increasing concentrations of ABT-199, DEX, and DAS for 48 h all three drugs reduced the number of viable cells in a dose-dependent manner with the triple-agent combination being most effective and killing almost all leukemic cells. However, drug-cytotoxicity was quite heterogeneous with ABT-199 most and less effective in P564 and R610 cells, respectively (P564 46% and R610 91% viable cells at 1 µM ABT199). In contrast, DAS cytotoxicity was highest in R610 cells and only mild in P564 cells (R610 50% and P564 94% viable cells at 100 nM dasatinib), whereas sensitivity of L4967 cells to all three drugs was intermediate (Fig. 5a). Nevertheless, cytotoxicity of the triple combination was synergistic in all three PDX-models with CI of 0.13, 0.10, and 0.11 for P564, R610 and L 4967 cells, respectively.

We next evaluated the effects of DEX and DAS treatment on MOMP induction by ABT-199. BV173 cells and cells from the three primografts were cultured in the presence or absence of DEX and DAS for 48 h before MOMP induction by ABT-199 was measured in the short-term TMRE-Assay (Fig. 5b). In all four models, pre-incubation with DEX/DAS significantly enhanced ABT-199 induced MOMP induction corresponding to the synergistic cytotoxicity of these three drugs.

Finally, we transplanted P564, L4951, and L4967 cells into NSG mice and monitored survival and leukemic engraftment by either flow cytometric analysis of human CD45+ cell kinetics in peripheral blood (P564 and L4967) or in vivo imaging (L4951). Due to limited cell numbers only two mice were transplanted with R610 cells (data not shown). Similar to the ex vivo data survival of untreated control mice was heterogeneous with all mice dying between 80 (rapid course) and 200 days (long course) (Fig. 5c). In contrast, all mice treated with the triple combination ABT-199/DEX/DAS survived the treatment period and beyond, and only two mice transplanted with P564 cells died about 100 days after the end of therapy. Figure S5B summarizes the survival data for all mice with rapid death due to untreated leukemia including both R610 mice demonstrating a significant survival benefit upon triple combination treatment. Flow cytometric analysis revealed clearance of human cells in all mice 4 weeks after start of treatment (Fig. 5d, P564 and L4967 cells). Similarly, no L4951 cells were detectable by in vivo imaging upon ABT-199/DAS/DEX treatment up to 2 weeks after end of treatment (Fig. S5C). In addition, blood smears were free of ALL blasts after 6 weeks of triple therapy in the P564 model (Fig. S5D). Taken together our data from biologically different primograft models support the concept of triple-agent therapy with ABT-199/DEX/DAS for efficient leukemia control.

## Discussion

We here show that a combination pharmacotherapy with ABT-199, dexamethasone and dasatinib is very effective and potentially curative in BCR-ABL + ALL. BH3-mimetics such as ABT-199 specifically target the BH3-binding groove in anti-apoptotic BCL2-family members thereby interrupting BH3-only protein binding [8, 9], while both steroids and TKIs enhance expression of mitochondrial BIM. TKIs additionally reduce expression of BCLXL and MCL1 [17]. Accordingly, we found synergistic cytotoxicity between ABT-199 on one hand and dexamethasone and TKI on the other in both cell line and BCR-ABL + ALL primograft models (see cartoon in S6).

Dexamethasone and the TKIs imatinib and dasatinib are important and crucial components of current pharmacotherapy for BCR-ABL + ALL. In our BV173 xenograft model triple-agent therapies of ABT-199, dexamethasone, and both TKIs were superior to double drug combinations both in terms of tumor growth and survival. However, dasatinib was much more effective than imatinib in combination with ABT-199 and dexamethasone with about 70% treatment free and MRD-negative long-term survival. In contrast, none of the mice treated with dual combinations of ABT-199/DAS or DEX/DAS became free of leukemia or survived after the treatment period for more than 2 weeks. Since these data indicated an unexpected curative potential the triple therapy was analyzed in more detail using different patients-derived primografts. ABT-199, DAS, and DEX act synergistically in all primografts tested in spite of heterogeneity in terms of cytotoxic response to ABT-199, dasatinib and/or dexamethasone alone. Furthermore, all mice engrafted with three different primografts not only survived the treatment period but became MRD-negative under ABT-199/DAS/DEX therapy demonstrating more generally the efficacy of this triple combination. Interestingly, dual combination therapy with ABT-199 and dasatinib has recently been described by Leonard et al. [19]. However, data on MRD negativity or treatment-free long-term survival have not been reported in that study. Accordingly, we propose the triple combination of ABT-199/DAS/DEX as potentially curative in several xenograft-models without excluding that other combinations or even dual therapies may be effective in individual models. Personalized approaches including quantification of MOMP induction by individual BH3-mimetics (see below) may help to optimize individual therapies in the future.

Dasatinib is a dual SRC/ABL TKI additionally targeting the LYN tyrosine kinase [20]. Therefore, LYN inhibition may contribute to the superiority of dasatinib as compared to imatinib in our experiments with inhibition of LYN providing an additional disturbance of survival signals beside those mediated by BCR-ABL. The observation that the cytotoxicity of both imatinib and dasatinib depends on BIM expression suggests that the additional dasatinib targets beside BCR-ABL may also converge on induction of mitochondrial apoptosis.

Our data also suggest a potential common mechanism of resistance with regard to induction of mitochondrial apoptosis for dexamethasone [21], BH3-mimetics, and TKIs in the absence of kinase domain mutations [22]. We demonstrate an uncoupling of BCR-ABL tyrosine kinase inhibition by imatinib from the induction of mitochondrial apoptosis in SUPB15 cells but synergism with and some “restoration” of imatinib cytotoxicity by ABT-199. The underlying molecular mechanism is currently unknown but may include differential expression of BCL2 or other anti-apoptotic proteins. Lesions in alternate proteins including SRC, JAK2, STAT3, and others have been proposed to explain treatment failure despite effective inhibition of BCR-ABL kinase activity [22]. Since ABT-199 cooperates with and reconstitutes imatinib cytotoxicity, we add loss of oncogene addiction at the level of MOMP induction as a potential mechanism of resistance. Along this line, a common polymorphism lacking the BIM-BH3-domain reduces therapeutic responses to both TKIs in CML and EGFR-inhibition in NSCLC [23]. For BH3-mimetics, mechanisms of resistance may include a shift in the dependency to different anti-apoptotic proteins, e.g., from BCL2 to BCLXL or MCL1, or mutations in the respective binding sites [24–26].

The different kinetics of cytotoxicity associated with ABT-199 and dexamethasone/TKI suggest that pulsatile ABT-199 therapy with reduced AUC as compared to continuous application may be particularly effective. Nevertheless, although pulsatile application of BH3-mimetics may indeed improve efficacy of combination therapies in vitro, in vivo imaging showed subclinical relapse of BCR-ABL + ALL in all mice treated this way. This could be in part due to the different application schedule, the reduction of ABT-199 dosage and/or the drug metabolism. However, it may still be worth to study modified time and dose schedules since a pulsatile application possibly reduces the frequency and severity of adverse effects. In particular, it will be interesting to study pulsatile administration of ABT-737/ABT-263 with regard to the induction of thrombocytopenia (which prevented further clinical development) in such settings.

In our luciferase transduced BV173 BCR-ABL + xenograft model we observed different clinical outcomes, namely treatment failure (dual combinations or triple combination of imatinib, ABT-199, dexamethasone), complete remission with early relapse (dose-modified combination with dasatinib, ABT-199, dexamethasone) or potential cure (“full dosis” triple combination of dasatinib, ABT-199, dexamethasone). These data suggest that rapid clearance of leukemia is necessary but not sufficient for cure. Rather high-dose treatment is required for long-lasting disease control and cure in addition to rapid tumor reduction. Our model therefore provides a useful tool to differentially study the impact of drug modifications on different clinical outcome parameters.

The induction of MOMP by BH3-mimetics can easily be visualized and quantitated on a single cell level in viable cells by flow cytometry using modified BH3 profiling techniques. This rapid assay provides a simple tool to measure cellular responses to various BH3-mimetics and may become particularly important to individually select the most effective BH3-mimetic, since MCL1 inhibitors and other BH3-mimetics are under clinical evaluation or may enter clinical use in the near future. For primary ALL cells, rapid quantification of MOMP induction will be especially beneficial since these cells are short-lived ex vivo. Once individual BH3-mimetics for BCL2, BCLXL, MCL1, BCLW, and/or BFL1 are clinically approved, individualized therapies based on MOMP-profiling should be feasible.

In primary leukemic cells, ABT-199 cytotoxicity is reduced in BCR-ABL+ as compared to BCR-ABL-ALL which may be due to reduced BIM expression. Here, we demonstrate that this inhibition can be reversed by combination therapy with dexamethasone and dasatinib. Recent evidence published by Mueschen and colleagues suggests that B-cell receptor signals or substitutes of them (e.g. provided by the BCR-ABL tyrosine kinase) have to be well balanced in B-lineage ALL [27]. For example, PTEN inhibitors, SYK activators and even fasting (via altered leptin-receptor signaling) can inhibit expansion of ALL cell populations indicating that the survival/apoptosis balance can be disturbed by several means [27–31]. According to our data, BH3-mimetics in combination with dexamethasone and dasatinib might be most suitable to induce mitochondrial apoptosis in BCR-ABL + ALL pointing to induction of mitochondrial apoptosis as a privileged therapeutic target in ALL cells.

In summary, our data provide rationally designed targeted, chemotherapy-free and potentially curative combination therapy with BH3-mimetics for BCR-ABL + ALL. Clinical trials are warranted and should include prospective testing of MOMP induction by BH3-mimetics. Due to its high anti-leukemic activity the chemotherapy-free and oral regimen with ABT-199, dasatinib and dexamethasone may be particularly useful for the large group of elderly patients not suitable for allogeneic stem cell transplantation.

## Supplementary information

Supplemental material
