# Supplementary material for: Optimized induction of mitochondrial apoptosis for chemotherapy-free treatment of BCR-ABL+acute lymphoblastic leukemia
Source: Leukemia. 2018 Dec 13;33(6):1313–23. doi: 10.1038/s41375-018-0315-6 (PMC6756054; doi:10.1038/s41375-018-0315-6)
Supplement: Supplementary file 1 — Supplemental material [file 41375_2018_315_MOESM1_ESM.pdf]

Supplementary Materials

Patient Materials

BM and PB samples were collected from newly diagnosed BCR-ABL-positive and negative B-lineage ALL, respectively (see Table S1). CML CD34$^+$ cells were purified to 95% or more by magnetic cell sorting (MACS, Miltenyi Biotech, Bergisch-Gladbach, Germany). PB samples from healthy volunteers served as controls. BM and PB samples from newly diagnosed BCR-ABL-positive and negative B-lineage ALL and healthy volunteers, and CML CD34$^+$ were obtained with written informed consent from the Ethics Committee of the Hannover Medical School. Primograft samples were derived by passaging individual primary patient samples through NSG mice.

Isolation and culture of human MSCs from bone marrow

For studies involving human bone marrow ethical approval from the ethical committee of Hannover Medical School was obtained. Samples were collected in accordance with the Declaration of Helsinki after written informed consent of the respective donors. Bone marrow samples were obtained during resection of the femoral head for implantation of a total hip arthroplasty in otherwise healthy donors. Human MSCs were isolated from these samples by density gradient centrifugation and subsequent plastic adhesion of mononuclear cells and cultured as described (Jungwirth N. et al., Mesenchymal Stem Cells Form 3D Clusters Following Intraventricular Transplantation. J Mol Neurosci. 2018;65(1):60-73. doi: 10.1007/s12031-018-1070-x). MSC preparations were routinely frozen in passage 2 (here: 11 days after surgery) with 95 % (v/v) fetal calf serum/ 5 % (v/v) dimethylsulfoxide and stored at – 140 °C.

Co-culture of ALL cells on MSC

MSC were seeded at a density of 10^4 cells/cm^2 in MSC media 48 hours prior to adding B-ALL cells. B-ALL cells were seeded onto MSC at a density of $1x10^6$ cells/ml in SFEM II medium

(Stemcell technologies, Cambridge, UK) supplemented with 20% FCS, 20 ng/ml recombinant IL3 (Peprotech, Hamburg, Germany), 10 ng/ml recombinant IL7 (Peprotech, Hamburg, Germany) and 1% penicillin/streptomycin. Non-adherent cells present in supernatant medium were collected followed by trypsination and collection of the adherent cell fraction, which contains both MSCs and adhered ALL cells. After passing through a 15 µm filter (pluriSelect Life Science, Leipzig, Germany), viable ALL cells were counted by trypan blue exclusion, resuspended in fresh SFEM II medium supplemented with IL-3, IL-7 and 20% FCS and seeded onto fresh MSC.

Cell Culture

BV173, SUPB15, Tom-1 (American Type Culture Collection, ATCC) are lymphoid cell lines carrying the BCR-ABL t(9;22) translocation. 697, NALM-6, and RS4;11 (ATCC) are BCR-ABL negative B cell precursor leukemias. The lymphoid and myeloid cell lines were cultured in RPMI 1640 supplemented with 10% FCS and 1% penicillin/streptomycin.

Pharmacologic agents

ABT-199 and Dexamethasone (DEX) were purchased from Selleck Chemicals and solubilized in dimethyl sulfoxid (DMSO) or PBS to 10 mM stock, respectively, and then supplemented to the culture medium at the required concentration. Imatinib mesylate (IM) was purchased from Sigma and dissolved in PBS to create a 1 mM stock. Dasatinib (DAS) and WEHI-539 were purchased from Santa Cruz Biotechnology and ApexBio, respectively and solubilized in DMSO at 10 mM stock. Doxorubicin hydrochloride (Sigma) was dissolved in PBS at 1 mM stock.

Drug treatment

PDX cells were co-cultured in black frame and clear flat-bottom 96-well plates (Thermofisher, Darmstadt, Germany) seeded with 10,000 MSCs per well 24 hours prior to treatment at a

B-ALL seeding density of $1x10^6$ cells/ml in SFEM II media. B-ALL cells were treated with increasing concentrations of ABT-199, Dexamethasone and Dasatinib alone or in combination in fixed ratios for 48h following Live/Dead Cell Viability Assays (Thermofisher, Darmstadt, Germany). Microscopic pictures were taken and the number of viable and dead cells was counted using a Axio Vert microscope (Zeiss, Jena, Germany). Drug combination indices were calculated using CompuSyn Software.

Construction of lentiviral vectors

shRNAs corresponding to position 37 to 55 of the human *BIM* gene (Gene bank accession no. NM_138621), to position 675 to 693 of the human *BID* gene (Gene bank accession no. NM_197966), lentiviral transgene plasmids pdc-SEW, and shRNA controls were cloned as described(32). The numbering of the first nucleotide of the shRNA refers to the ATG start codon. Lentiviral constructs encompassing the shRNAs encode GFP (green fluorescent protein) as a reporter gene. The preparation of recombinant lentiviral supernatants and lentiviral transductions were performed as described earlier(33). Transduction efficacy based on GFP-expression was > 90% in all experiments.

RNA isolation, and qRT-PCR

Total RNA from cell lines was prepared using Trizol (Invitrogen). For qRT-PCR of mRNAs, cDNA synthesis was performed with 1 µg of total RNA digested with *DNaseI* and subjected to TaqMan-based (Applied Biosystems) gene expression profiling following the manufacturer's protocol. Primer/probe assays for human *BIM* (Hs00375807_m1), human *BID* (Hs01026792_m1), and *B2M* (Hs99999907_m1) were purchased from Applied Biosystems. *B2M* served as an internal control. Real-time PCR was performed using an ABI7500 cycler (Applied Biosystems).

Apoptosis Assay

1 x 10^5 cells were stained with 10 µg/ml Propidium iodide (Sigma-Aldrich, Hamburg, Germany). Apoptotic cells were measured with a FACS Calibur flow cytometer, and data were analyzed with Cell-Quest Pro software (BD). CI values were calculated using CompuSyn software. To calculate the IC50-values, the cell lines were plated in 96-well plates at 1x10^5 cells/ml in the presence of increasing concentrations of ABT-199 as indicated. The number of viable cells was determined 48 hours later by PI staining. The number of PI+ cells was plotted against the concentration of ABT-199 and IC_{50}-values were calculated using GraphPad Prism software.

Immunoblotting, Co-immunoprecipitations

Whole cell lysates were prepared with lysis buffer (20 mM HEPES, pH 7.5, 0.4 M NaCl; 1 mM EDTA, 1 mM EGTA, 1 mM DTT) supplemented with mini complete protease inhibitor cocktail tablet (Roche Diagnostics, Mannheim, Germany) and separated by sodium dodecyl sulphate-polyacrylamide gel electrophoresis (SDS-PAGE), transferred to Hybond enhanced chemiluminescence (ECL) nitrocellulose membrane (Amersham Bioscience, Uppsala, Sweden) and membranes were incubated with the following antibodies according to the manufacturer's protocol: anti-BCLW (cs2724), anti-MCL1 (cs4572), anti-BIM (cs2933), anti-BID (cs2002), anti-PUMA (cs4976), anti-BAK (cs 12105), anti-BAX (cs2772), anti-PARP (cs9542), anti-GAPDH (cs2118), anti-pERK (cs9101), and anti-pBIM (cs4581) from Cell Signaling Technology; anti-ERK2 (sc-154), and p-TYR (sc-7020) from Santa Cruz Biotechnology; anti-BAD (62465) from Abcam; and anti-BCL2 (551051) from Becton Dickinson. Chemiluminescence was used for visualization using the ECL Western blotting detection reagents (PerkinElmer) according to the manufacturer. Densitometric analysis of x-ray films was performed using VersaDoc 3000 Imaging system (Bio-Rad) and Image Lab software version

5.0 (Bio-Rad). The intensity ratio of the protein of interest band to the GAPDH band (loading control) was calculated to measure changes in protein levels.

For co-immunoprecipitation studies, BV173 or SUPB15 cells were lysed in CHAPS-buffer (50 mM Tris-HCl [pH 7.4], 150 mM NaCl, 1 mM EGTA, 1 mM EDTA, 1% CHAPS) supplemented with mini complete protease inhibitor cocktail tablet on ice for 30 min. Input protein was precleared and then precipitated with one of the following antibodies: anti-BCL2 (AW604, Millipore), anti-BCLXL (cs2762, Cell Signaling), anti-MCL1 (MABC43, Millipore) or anti-BIM (MAB17001, Millipore) precoupled to protein A/G magnetic beads (Bio-Rad) at 4°C. After 18 h, immuno-complexes were recovered, washed, and resuspended in sample buffer and subjected to western blot analysis. Quantitative densitometric analysis of co-immunoprecipitations was performed by chemiluminescence imaging of immunoblots using ChemiDoc MP Imaging system (Bio-Rad).

Animal experiments

Leukemic BV173 cells (BV173-SLIEW) or PDX cells ($1x10^6$) were adoptively transplanted into recipient NSG (NOD.Cg-Prkdcscid Il2rgtm1WjI/SzJ) mice intravenously. DEX (1 mg/kg) and IM (20 mg/kg) (Sigma-Aldrich, St. Louis, MO) were treated by oral gavage 5 days per week or in combination with ABT-199 (20, 50 and 75 mg/kg) in a vehicle consisting of 60% Phosal, 30% PEG 400 and 10% Ethanol with a treatment delay of minimum 2 hours. The combination of DAS (10 mg/kg) and DEX was treated orally in 0.1M sodium citrate or in combination with ABT-199 (20 mg/kg) with a treatment delay of minimum 2 hours. Detailed dosing information is indicated in the figure legends. Tumor burden was assessed either by immunophenotyping of peripheral blood samples using human CD45-APC /CD19-APC antibodies or by *in vivo* bioluminescent assay. D-Luciferin (1 mg/mouse) (AppliChem, Darmstadt, DE) was provided intraperitoneally. Life imaging of tumor growth was detected weekly by using IVIS Lumina II (Caliper Life Sciences, Hopkinton, MA). Survival was monitored daily. Living Image 4.0 software was used to analyze the bioluminescence radiance. Female

mice used in the experiments were 8 to 10 weeks old and were randomly allocated to each group. All animal studies were in accordance with the German animal protection law and with the European Communities Council Directive 86/609/EEC and 2010/63/EU for the protection of animals used for experimental purposes. All experiments were approved by the Local Institutional Animal Care and Research Advisory Committee and permitted by the local authority, the Niedersächsisches Landesamt für Verbraucherschutz und Lebensmittelsicherheit [No. 33.14-42502-04-16/2217] and [33.12.42502-04-17/2494].

References

32. Scherr M, Battmer K, Ganser A, and Eder M. Modulation of gene expression by lentiviral-mediated delivery of small interfering RNA. *Cell cycle.* 2003;2(3):251-7.
33. Venturini L, Battmer K, Castoldi M, Schultheis B, Hochhaus A, Muckenthaler MU, Ganser A, Eder M, and Scherr M. Expression of the miR-17-92 polycistron in chronic myeloid leukemia (CML) CD34+ cells. *Blood.* 2007;109(10):4399-405.
34. Rehe K, Wilson K, Bomken S, Williamson D, Irving J, den Boer ML, Stanulla M, Schrappe M, Hall AG, Heidenreich O, et al. Acute B lymphoblastic leukaemia-propagating cells are present at high frequency in diverse lymphoblast populations. *EMBO molecular medicine.* 2013;5(1):38-51.

Supplemental Figure 1

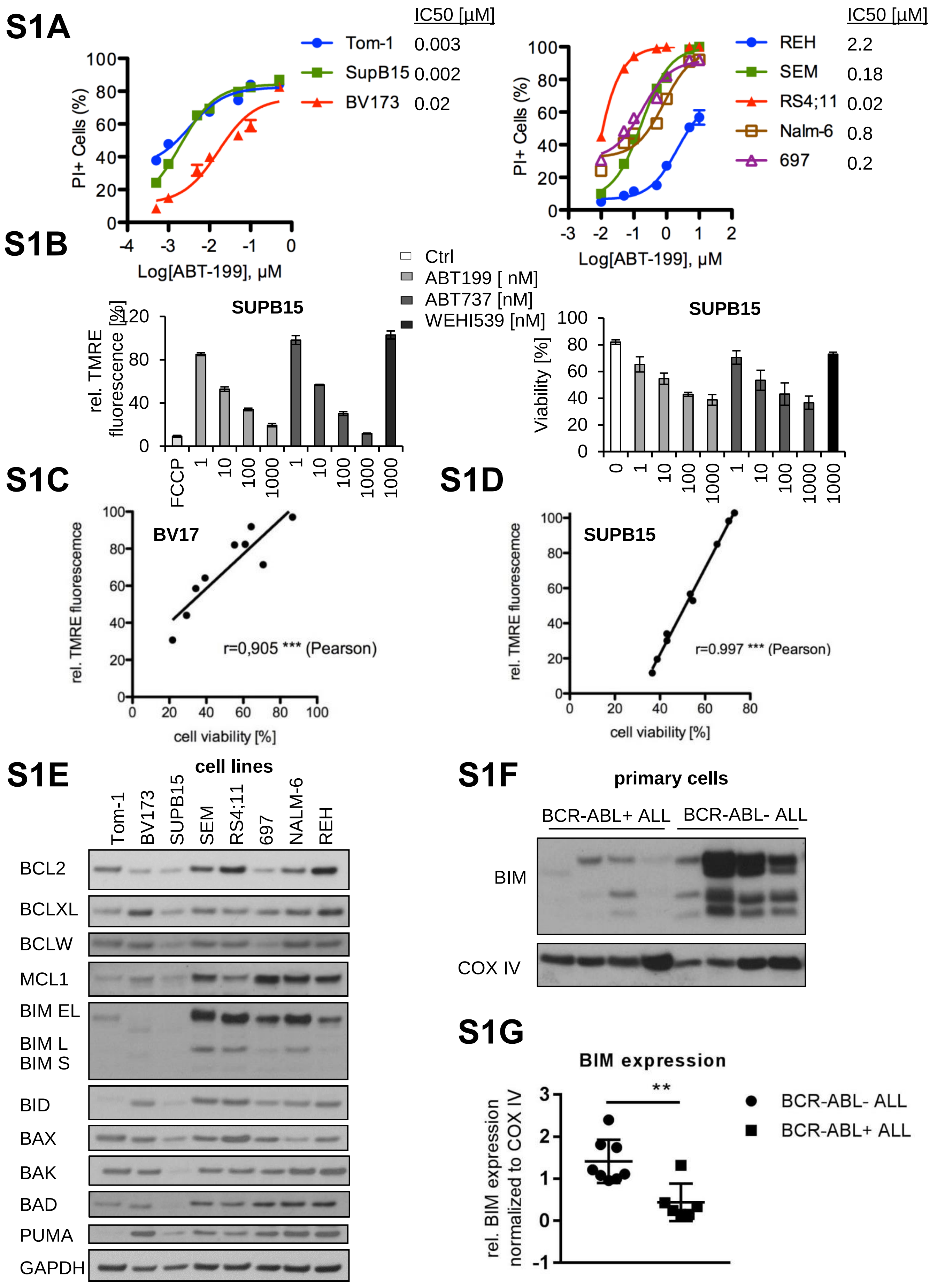

S1H

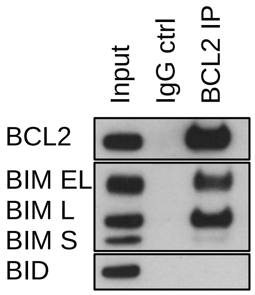

S1I

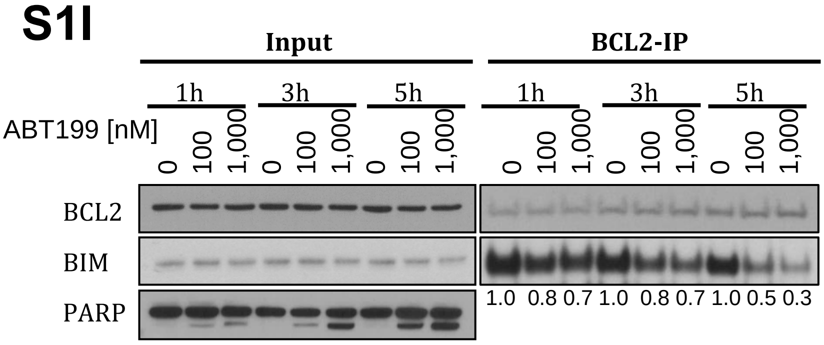

S1J

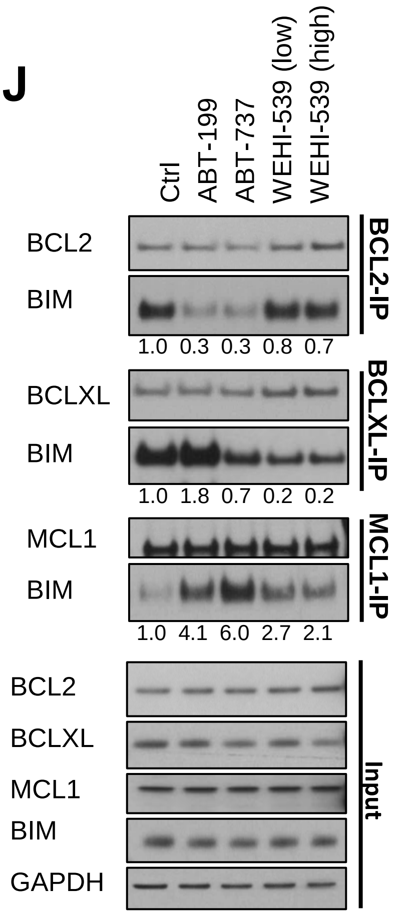

S1K

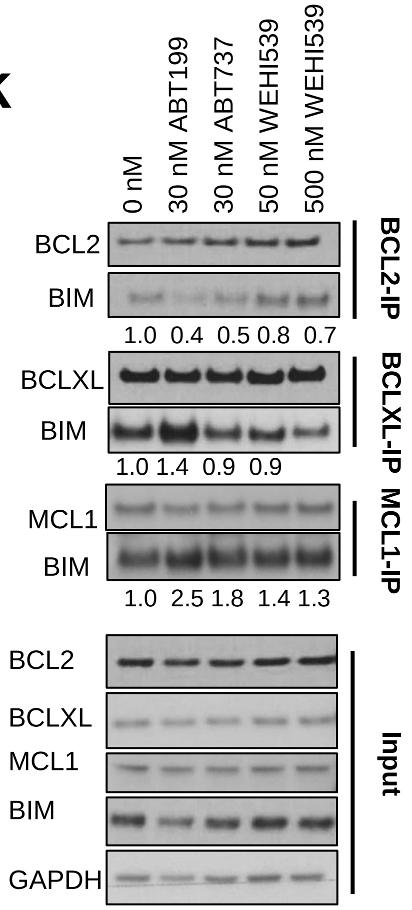

S1M

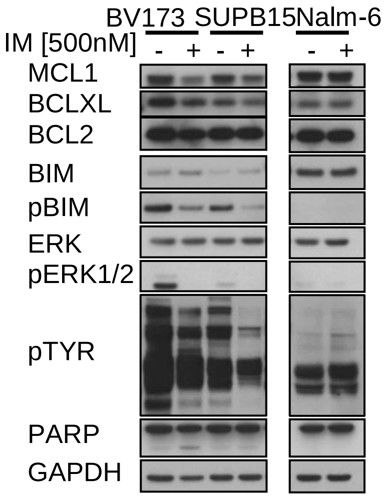

S1L

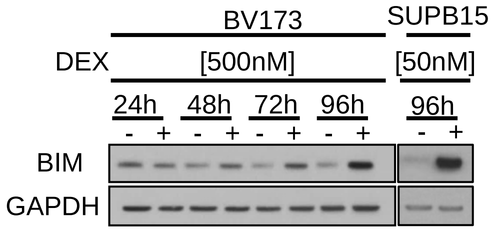

Supplemental Figure 1 (a) IC_{50} analysis of BCR-ABL-positive (left) and BCR-ABL-negative (right) ALL cell lines. Cell death was assessed by propidium iodide (PI) staining after treatment with increasing concentrations of ABT-199 for 48 hours. (**b**) (left) SUPB15 cells were treated with various concentrations of ABT-199, ABT-737 and WEHI-539 for 3 hours followed by staining with TMRE to determine MOMP of viable cells by flow cytometry. Treatment with the ionophore FCCP served as positive control, and fluorescence of untreated cells was set as 100%. (right) PI staining to monitor cell viability was performed by flow cytometry after an additional 21 hours. Values are expressed as means ± SD. (n=3). **(c, d)** The Pearson test was used to determine the correlation coefficient between MOMP induction in BV173 (**c**) or SUPB15 cells (**d**) as defined by relative TMRE fluorescence and apoptosis as defined by PI-staining (***p<0.001). (**e**) Representative western blot analysis of protein expression of BCL2-family members in BCR-ABL-positive and -negative cell lines. **(f)** Western blot analysis of BIM expression in primary BCR-ABL-positive and BCR-ABL-negative ALL cells. COX IV served as loading control. (**g**) Quantification of BIM protein expression in primary BCR-ABL-positive and BCR-ABL-negative ALL cells. Relative expression of BIM was normalised to COX IV expression. P-value was calculated by Student's *t*-test (**$p < 0.01$) (**h**) Cellular lysates of BV173 cells were immunoprecipitated using anti-BCL2 or anti-IgG antibodies and subjected to western blot analysis for BCL2, BIM and BID expression. (**i**) BV173 cells were treated with increasing concentrations of ABT-199 for the indicated periods of time. Whole cell lysates and BCL2-immunoprecipitates were immunoblotted with anti-BCL2, anti-BIM and anti-PARP antibodies as indicated. Densitometric analysis was performed as compared to untreated controls at the respective time points. **(j)** BV173 cells were treated with ABT-199 (100 nM), ABT-737 (100nM), and WEHI-539 (low=50 nM, high=500 nM) for 5 hours and cellular lysates were immunoprecipitated with anti-BCL2, anti-BCLXL, and anti-MCL1 antibodies. Cellular lysates and immunoprecipitates were subjected to western blot analysis using anti-BCL2-, anti-BCLXL-, anti-MCL1-, and anti-BIM-antibodies. GAPDH was used as loading control. All western blots are representative for three independent experiments **(k)** SUPB15 cells were treated with ABT-199, ABT-737, and WEHI-539 at the indicated concentrations for 5 hours and cellular lysates were immunoprecipitated with anti-BCL2, anti-BCLXL, and anti-MCL1 antibodies. Cellular lysates and immunoprecipitates were subjected to western blot analysis using anti-BCL2-, anti-BCLXL-, anti-MCL1-, and anti-BIM-antibodies. GAPDH was used as loading control. **(l)** BV173 and SUPB15 cells were treated with dexamethasone for the indicated periods of time. Cellular lysates were subjected to western blot analysis using anti-BIM and anti-GAPDH antibodies. **(m)** BV173, SUPB15 and Nalm-6 cells were treated with 500 nM IM for 16 hours followed by western blot analysis using the indicated antibodies. All western blots are representative for three independent experiments.

Supplemental Figure 2

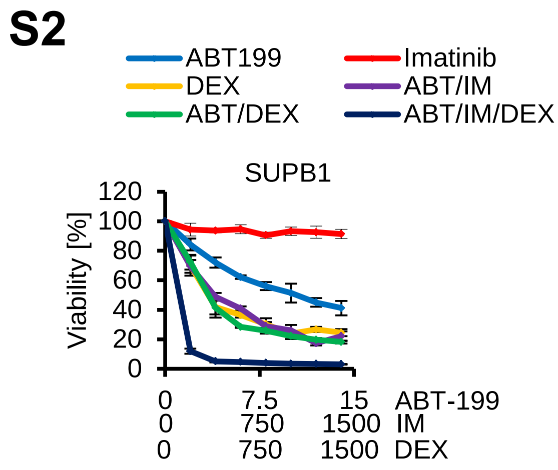

Supplemental Figure 2: SUPB15 cells were treated with ABT-199, IM, DEX alone or in combination at the indicated concentrations at fixed ratios for 48 hours followed by PI staining. Values are expressed as means ± SD (n=3). Combination indices were calculated using the Chou Talalay method. CI values<1 were considered as synergistic drug interaction. CI(ABT/DEX):1.31, CI (ABT/IM)=0.40, CI (ABT/DEX/IM)=0.03

Supplemental Figure 3

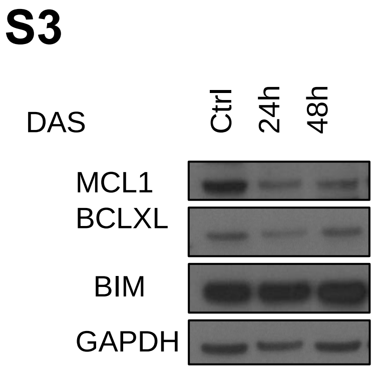

Supplemental Figure 3 Western blot analysis for BIM, BCLXL and MCL1 expression of BV173 cells treated with 10 nM DAS for the indicated periods of time. GAPDH served as loading control.

Supplemental Figure 4

S4A

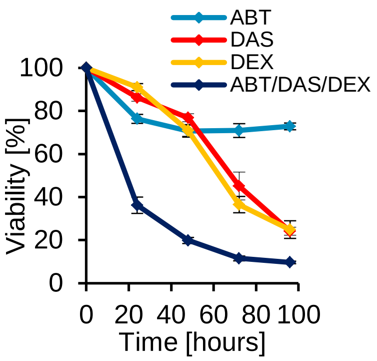

S4B

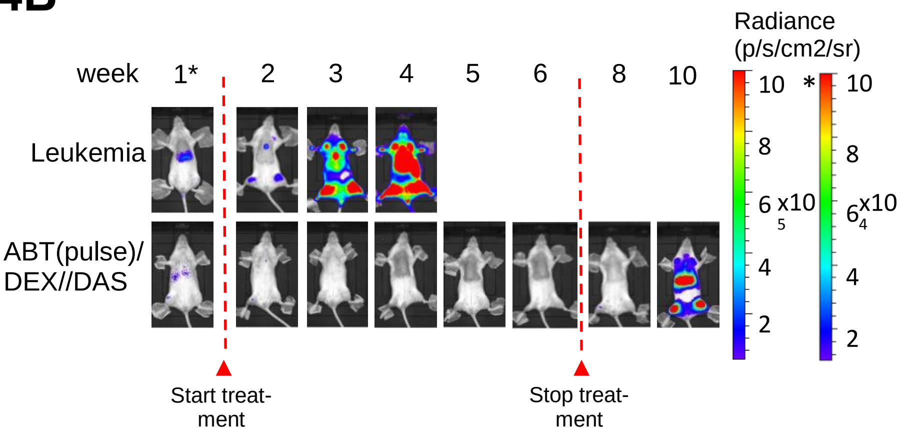

Supplemental Figure S4: (a) BV173 cells were treated with ABT-199, DAS, DEX alone or in combination at 20 nM over a period of 96 hours. PI staining was performed at the indicated time points. **(b)** NSG recipients received $1x10^6$ BV173 cells intravenously. Tumor proliferation was monitored by using *in vivo* bioluminescent IVIS assay. Treatment started one week after tumor inoculation. Representative IVIS results for week 1-10 of recipients treated with DEX (1 mg/kg) and DAS (10 mg/kg) by oral gavaging 5 days per week and pulsatile therapy with ABT-199 (constant dose of 20 mg/kg) on the 4th day (96 hours) of the weekly treatment cycle are shown. Treatment was stopped after week 6.

Supplemental Figure 5

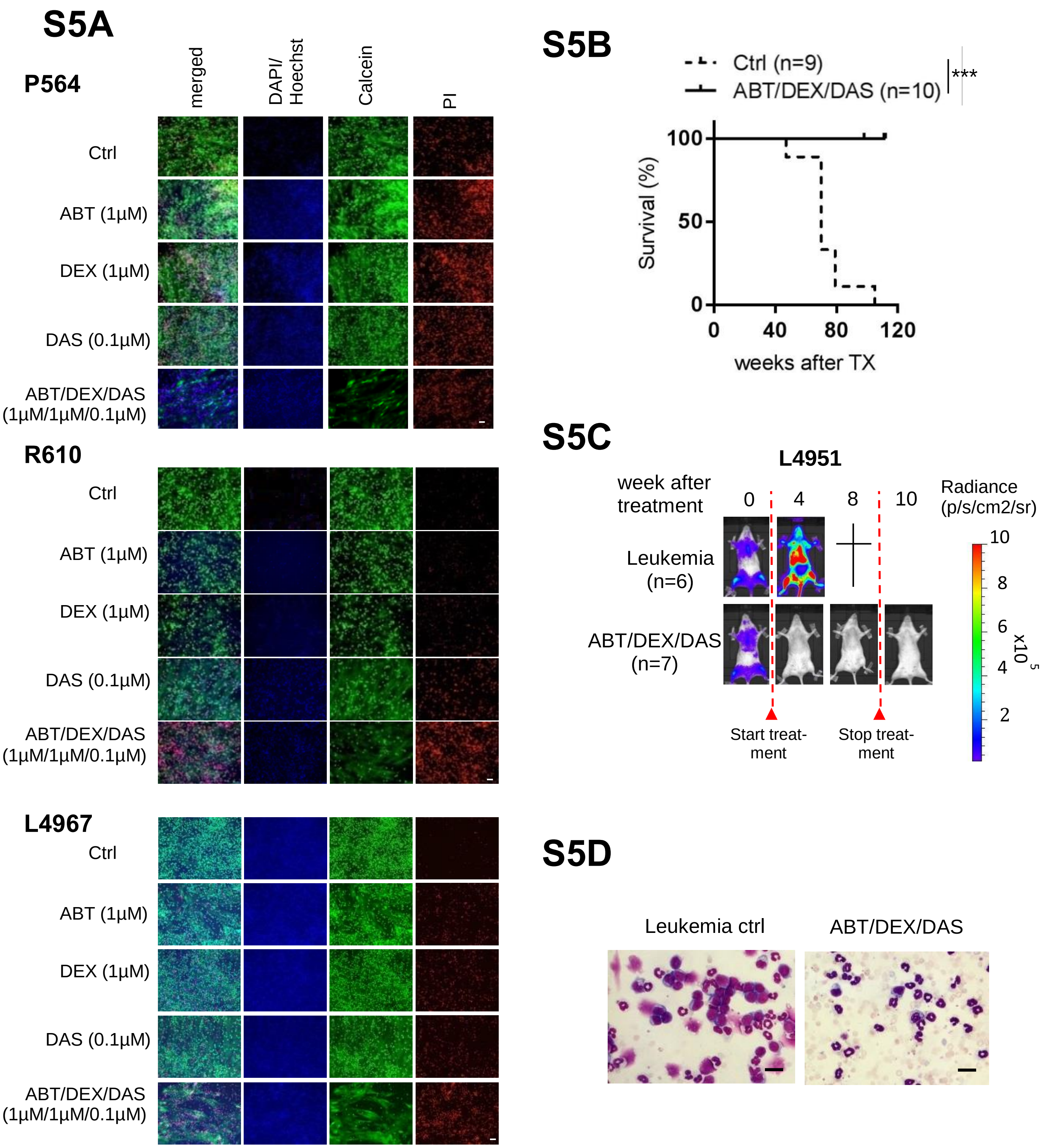

Supplemental Figure S5: (a) Resprestentative fluorescence microscopic analysis of patient-derived P564 (upper), R610 (middle) and L4967 (lower) cells. Cells were plated 24 hours prior to drug treatment onto primary MSCs. Co-cultures were treated with increasing concen-

trations of ABT-199, DEX and DAS alone or in combination with fixed ratios. Fluorescence microscopic analysis of Calcein AM/PI/DAPI staining was performed to determine apoptotic cell death (green Calcein staining of viable cells, red PI staining of apoptotic cells, blue DAPI/Hoechst staining of nuclei) Scale bars indicate 50 µm. (**b**) Cumulative survival of NSG recipients transplanted with rapidly lethal patient-derived leukemia (R610, L4951 and L4967) is summarized. (**c**) Tumor cell kinetics of L4951 primograft was monitored by using *in vivo* bioluminescent IVIS assay. Respresentative IVIS results for week 0-10 after treatment start. (**d**) Representative morphology on cytospin preparations of untreated and ABT/DEX/DAS treated NSG mice transplanted with patient-derived P564 cells four weeks after treatment start.

Supplemental Figure 6

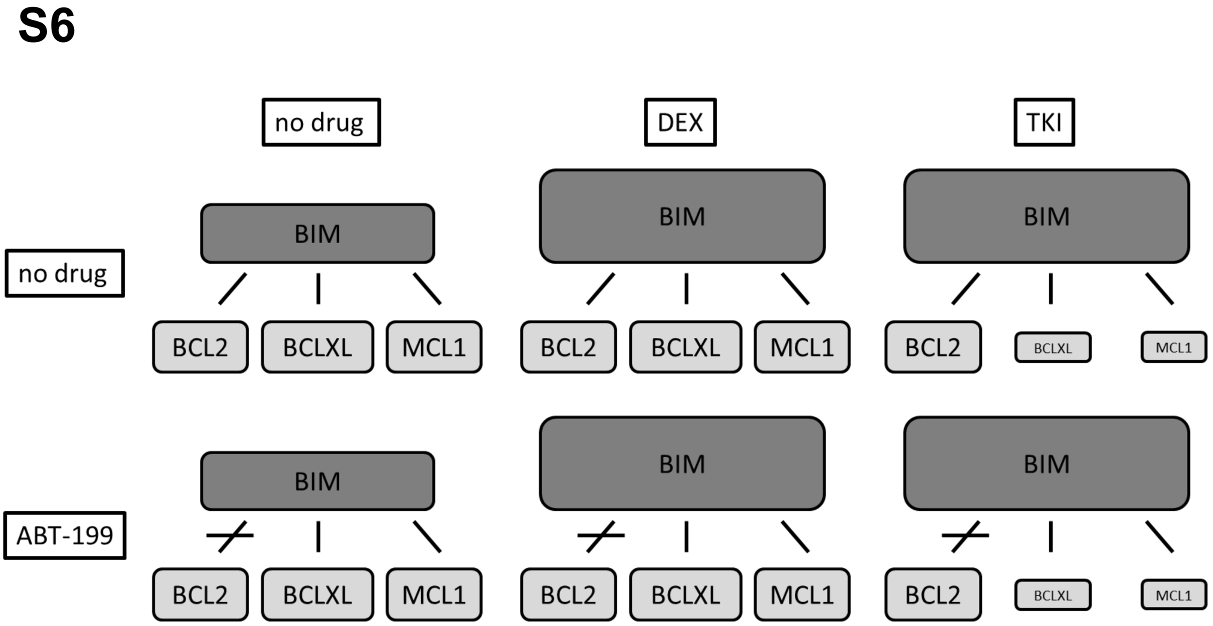

Supplemental Figure S6: Cartoon depicting drug interactions between ABT-199, dexamethasone and TKIs in BCR-ABL+ ALL cells. Pro-apoptotic BIM may bind to BCL2, BCLXL and MCL1 (left upper panel) whereas ABT-199 interrupts the binding of BIM to BCL2 (left lower panel). Dexamethasone enhances BIM expression (middle upper panel) which is again released from BCL2 by ABT-199 (middle lower panel). TKIs both enhance BIM expression and reduce MCL1 and BCLXL expression (upper right panel). ABT-199 releases BIM from BCL2 (lower right panel). BIM actives BAK and/or BAX leading to mitochondrial outer membrane permeabilisation (MOMP) and subsequent apoptosis in ALL cells.

Supplemental Table 1

sample	diagnosis	Cytogenetics/molecular genetics	age at presentation	gender	blast count (%)
1	pre-B-ALL	n.a./ bcr-abl neg	69	male	80
2	pre-B-ALL	n.a./ bcr-abl neg	20	male	11
3	pre-B-ALL	normal/ bcr-abl neg	56	female	20
4	pre-B-ALL	t(12;21)/ bcr-abl neg	19	male	45
5	pro-B-ALL	t(4;11) bcr-abl neg	73	female	91
6	pre-B-ALL	normal/ bcr-abl neg	19	male	71
7	pre-B-ALL	n.a./ bcr-abl neg	65	female	95
8	pre-B-ALL	n.a./ bcr-abl neg	82	female	n.a.
9	pre-B-ALL	normal/ bcr-abl neg	75	female	51
10	pre-B-ALL	normal/ bcr-abl neg	53	male	70
11	pre-B-ALL	+13/ bcr-abl neg	25	male	50
12	pre-B-ALL	n.a./ bcr-abl neg	85	male	98
13	pre-B-ALL	t(12;21)/ bcr-abl neg	18	female	85
14#	pre-B-ALL	45, XX, t(1;16)(q21;q22), -7, t(9;22)/ bcr-abl: p190	59	female	54
15	pre-B-ALL	t(9;22)/ bcr-abl: p190	19	female	56
16	pre-B-ALL	t(9;22)/ bcr-abl: p210	49	male	90
17	pre-B-ALL	t(9;22)/ bcr-abl: p190	47	female	72
18	pre-B-ALL	t(9;22)	73	male	95
19	pre-B-ALL	t(9;22)/ bcr-abl: p210	73	female	17
20*	pre-B-ALL	t(9;22)/ bcr-abl: p210	59	female	90

Supplemental Table S1: Clinical data for ALL patients at presentation: primary cells and primografts

primograft R610; * primograft P564, n.a. : not available ; Primografts L4591 and L4567 have recently been described(34).
